# Prognostic factors analysis for oral cavity cancer survival in the Netherlands and Taiwan using a privacy-preserving federated infrastructure

**DOI:** 10.1038/s41598-020-77476-2

**Published:** 2020-11-25

**Authors:** Gijs Geleijnse, RuRu Chun-Ju Chiang, Melle Sieswerda, Melinda Schuurman, K. C. Lee, Johan van Soest, Andre Dekker, Wen-Chung Lee, Xander A. A. M. Verbeek

**Affiliations:** 1 Netherlands Comprehensive Cancer Organisation (IKNL), Godebaldkwartier 419, 3511 DT Utrecht, The Netherlands; 2 Institute of Epidemiology and Preventive Medicine, College of Public Health, National Taiwan University and Taiwan Cancer Registry, Taipei, Taiwan; 3 Biomedical Technology and Device Research Laboratories, Industrial Technology Research Institute (ITRI), Hsinchu, Taiwan; 4 Department of Radiation Oncology (MAASTRO), GROW School for Oncology and Developmental Biology, Maastricht University Medical Centre, Maastricht, The Netherlands

**Keywords:** Cancer epidemiology, Information technology, Software

## Abstract

The difference in incidence of oral cavity cancer (OCC) between Taiwan and the Netherlands is striking. Different risk factors and treatment expertise may result in survival differences between the two countries. However due to regulatory restrictions, patient-level analyses of combined data from the Netherlands and Taiwan are infeasible. We implemented a software infrastructure for federated analyses on data from multiple organisations. We included 41,633‬ patients with single-tumour OCC between 2004 and 2016, undergoing surgery, from the Taiwan Cancer Registry and Netherlands Cancer Registry. Federated Cox Proportional Hazard was used to analyse associations between patient and tumour characteristics, country, treatment and hospital volume with survival. Five factors showed differential effects on survival of OCC patients in the Netherlands and Taiwan: age at diagnosis, stage, grade, treatment and hospital volume. The risk of death for OCC patients younger than 60 years, with advanced stage, higher grade or receiving adjuvant therapy after surgery was lower in the Netherlands than in Taiwan; but patients older than 70 years, with early stage, lower grade and receiving surgery alone in the Netherlands were at higher risk of death than those in Taiwan. The mortality risk of OCC in Taiwanese patients treated in hospitals with higher hospital volume (≥ 50 surgeries per year) was lower than in Dutch patients. We conducted analyses without exchanging patient-level information, overcoming barriers for sharing privacy sensitive information. The outcomes of patients treated in the Netherlands and Taiwan were slightly different after controlling for other prognostic factors.

## Introduction

The difference in incidence of oral cavity cancer (OCC) between both the Netherlands and Taiwan is striking. Taiwan has one of the world’s highest incidence rates of OCC^1^. In 2016, 5116 patients were diagnosed with OCC with a standardized incidence rate of 13.8 cases per 100,000 population. Men in Taiwan are at 10.8 times higher risk for OCC than women^2^. Contrary to Taiwan, OCC in the Netherlands is a rare disease with an annual incidence of approximately 900 cases (or 5.5/100,000 inhabitants)^3^. Changes in incidence, mortality and survival may reflect changes in risk factors, diagnostics, clinicopathological factors, and treatment^4,5^. To be able to provide high standards of care, the treatment of head and neck tumours in the Netherlands is centralized within 14 expertise centres. Expertise and patients’ characteristics may result in survival differences between these different geographical areas. Also, prognostic factors for OCC survival may have differential effects in patients of these two countries. However due to regulatory restrictions, patient-level analyses where data is shared between these countries is unfeasible.

With the implementation of the General Data Protection Regulation (GDPR) in the European Union, cancer registries are amidst an on-going debate on its implications^6^. The GDPR may be one of the arguments for data processing entities such as cancer registries to be reticent in data sharing initiatives. In particular, the GDPR poses restrictions on data sharing with parties outside the European economic area, including Taiwan. In collaborations such as Eurocare, Globocan and RARECARE, cancer registries are sharing patient-level data to facilitate large scale international epidemiological research^7–9^. In such international studies, the patient record data are typically delivered to a trusted organization, responsible for processing the pooled data. Said regulations and privacy concerns pose a threat to the continuation of these initiatives.

Innovations in information technology have created an alternative to the traditional pooling of data. Ohno-Machado and colleagues developed a series of algorithms “building shared models without sharing data”, in order to compute regression models without record level data leaving the participating organizations^10–12^. Feasibility of federated privacy-preserving classification algorithms and survival analyses have been demonstrated using mathematical and experimental analyses^13–16^. Several other machine learning algorithms were created for “distributed learning” and successfully applied it to a number of studies in involving multiple radiotherapy centres using a commercial software application^17,18^. We developed an open source implementation of a federated privacy preserving data analysis platform^19,20^. Unlike other initiatives^21–23^, it offers a flexible open-source infrastructure that allows to deploy federated algorithms implemented in wide range of programming languages. Hence, the infrastructure allows to deploy existing algorithms as described in literature and combine them into a series of analyses. Also, it does not assume a prescribed data format, which makes it suitable for cancer registries.

In this work, we apply the federated privacy preserving data analysis platform to compare the prognostic factors for OCC survival between Taiwan and the Netherlands.

## Methods

### Data

The Taiwan Cancer Registry (TCR) is a national population-based cancer registry system established in 1979. Data from Taiwanese patients with newly diagnosed malignancies in hospitals with 50 or more beds are mandatory reported to the TCR. Details of the history, objectives, and activities of the TCR have been well-documented^24^. With its high data quality and completeness (approximately 98%), the TCR is also one of the highest-quality cancer registries in the world^25^.

The Netherlands Cancer Registry (NCR) is a nationwide registry in which all newly diagnosed malignancies in the Netherlands are documented. It has a nationwide coverage since 1989. The main source of notification of the NCR is the automated nationwide network and registry of histo- and cytopathology (PALGA) and it is complemented by other sources such as the National Registry of Discharge Diagnosis. After notification, specially trained registry clerks routinely extract data on patients and tumour characteristics from patient’s medical records in all Dutch hospitals.

The dataset for each registry follows standard research protocols, and the selected variables are converted into defined code. All patients who underwent surgery for a diagnosis with an oral cavity squamous cell carcinoma (ICD-O Topography codes C00.3-5, C02, C03, C04, C05.0, C05.8, C05.9, C06 and morphology codes 8050-8089) between 2004 and 2016 were selected from the TCR and NCR. In case of multiple primary OCCs, only the first primary tumour was included in the study. In both registries, tumour topography, morphology and grade were coded according to the International Classification for Disease Oncology 3rd Edition-(ICD-O-3). For tumour stage, the Netherlands uses the Tumour Node Metastases (UICC TNM, 6th and 7th editions), whereas Taiwan adopts the AJCC 6th and 7th editions cancer staging system. However, the UICC and AJCC cancer staging systems are almost the same, so the staging data are comparable. Treatment was categorized in primary surgery and surgery with adjuvant radiotherapy and/or chemotherapy. Hospital volume was defined as the number of OCC surgeries performed in the centre where the patient was treated in the year of the patient’s diagnosis. Volume was divided into 3 categories (< 50, 50–99 and ≥ 100 surgeries/year). Survival was defined as the time from date of diagnosis to date of death or until the last date of follow-up. Data on vital status and date of death through linkage with the population death databases were collected up to January 31, 2019. This study was approved by Netherlands Cancer Registry’s Supervisory Committee (K18.098) and the National Taiwan University Hospital Research Ethnics Committee (201801116RINA).

### Federated infrastructure

To enable the privacy-preserving analysis of the Dutch and Taiwanese data, open source software was written to facilitate the analysis of local data and communication of aggregated statistics. We created a software infrastructure, where a server coordinates computing tasks and is connected via the internet to the computers (nodes) of the two organizations. The system conceptually consists of three components: a central server, multiple nodes and (software run by) a researcher. Each participating site runs a node that has access to the patient-level data and connects to the central server. The central server handles administrative tasks like authentication and authorization, and acts as a central point for communication between the nodes. Software run by researchers can upload "tasks", for example "compute the sums over all columns", to the central server, which are picked up by the nodes and executed. While tasks run on patient level data, the nodes only return aggregated data, no patient identifiable data is shared. Multiple tasks can be chained to create a script including more complex or iterative algorithms. Orchestration is then performed by software run on the researcher’s computer. A more detailed and technical description of the infrastructure as well as all open source software can be found at the website^19^.

### Statistical analysis

The means or frequencies of patient characteristics, treatment modalities and hospital volume were compared between countries. Chi-square test was used for analysing categorical variables. A federated version of the Cox proportional hazard algorithm with Breslow’s method for ties was implemented^19^. Mathematical decomposition of the algorithm and its soundness were demonstrated by Lu and colleagues^12^. Briefly, the nodes iteratively compute aggregated statistics based on the latest estimates of the hazard ratios (HRs) and the local registry data. Next, the aggregated statistics from the sites are combined to compute an updated estimation of the HRs. Finally, the estimation of the HRs has converged, the algorithm finishes. We also performed interaction analyses to assess whether the prognostic factors of OCC are different or have differential effects on survival between the Netherlands and Taiwan. *P* values for interaction are based on the likelihood ratio test of the interaction term between “country” and the respective prognostic factors.

Following Lu et al. we implemented the Newton–Raphson update to iteratively estimate the HRs for the selected covariates. This implementation is known to converge quickly (i.e. require few iterations), but it requires complex computations for each iteration. To restrict the complexity, we use a follow up period in years with one decimal rather than a period in days. In our analysis, the algorithm terminates when the difference between the sums of the previous and updated HRs after an iteration is less than 10^–8^.

## Results

### Patient characteristics

A total of 7766 and 33,867 newly diagnosed OCC cases with single primary tumour and receiving surgical treatment were recorded from 2004 to 2016 in the Netherlands and Taiwan, respectively (Table 1). In the Netherlands, the mean age was 63.9 years and among them, 44% were men. However, the mean age in Taiwan was 10 years younger (53.3 years) than the Netherlands and more than 91% of patients were men. The common sites of OCC in the Netherlands were floor of the mouth and gum (41.8%) and other/unspecified parts of tongue (41.2%); but in Taiwan, the common sites were buccal and other parts of mouth (44.8%) and other/unspecified parts of tongue (36.3%). Additionally, most patients in the Netherlands were treated in hospitals with the lowest hospital volume (< 50 oral surgery/year, 59%), while in Taiwan, nearly two-third of patients received treatment in hospitals with the highest hospital volume (≥ 100 oral surgery/year, 64%). Similarly, period of diagnosis, cancer stage, tumour grade, and treatment modalities between the two countries were all significantly different.

**Table 1:** Patients characteristics.

	Netherlands		Taiwan		*P* value
	Cases	%	Cases	%	
**Total**	7766	100.0	33,867	100.0	
**Age (average)**	63.9		53.3		
< 60 years	2709	34.9	24,493	72.3	
60–69 years	2542	32.7	6196	18.3	< .001
≥ 70 years	2515	32.4	3178	9.4	
**Gender**					
Male	4356	44.0	30,913	91.3	
Female	3410	56.0	2954	8.7	< .001
**Period of diagnosis**					
2004–2007	2148	30.2	7873	23.2	
2008–2011	2400	33.6	10,528	31.1	< .001
2012–2016	3218	36.2	15,466	45.7	
**Stage**					
I	3392	43.8	11,239	33.2	
II	1220	15.8	6918	20.4	
III	827	10.8	3946	11.7	
IVA	2208	28.1	10,269	30.3	< .001
IVB	64	0.8	969	2.9	
IVC	17	0.2	81	0.2	
Unknown	38	0.5	445	1.3	
Early stage	4612	59.4	18,157	53.6	
Advanced stage	3116	40.1	15,265	45.1	< .001
Unknown	38	0.5	445	1.3	
**Location**					
Mucosa of lip (ICD-O C003-005)	114	1.5	728	2.1	
Other/unspecified parts of tongue (ICD-O C02)	3215	41.2	12,282	36.3	
Floor of mouth and gum (ICD-O C03-04)	3234	41.8	5049	14.9	< .001
Hard palate (ICD-O C050, C058-059)	117	1.4	636	1.9	
Buccal and other parts of mouth (ICD-O C06)	1086	14.1	15,172	44.8	
**Grade**					
Well differentiated	1183	15.1	11,285	33.3	
Moderately differentiated	4084	52.1	17,677	52.2	< .001
Poorly or undifferentiated	1075	14.3	2355	7.0	
Unknown	1424	18.5	2550	7.5	
**Treatment**					
Primary surgery	4876	63.0	18,570	54.8	
Surgery with radiotherapy and/or chemotherapy	2890	37.0	15,297	45.2	< .001
**Hospital volume (oral cavity surgeries/year)**					
< 50	4466	59.3	5272	15.6	
50–99	2560	34.7	6992	20.6	< .001
≥ 100	740	6.0	21,603	63.8	

### Univariable analyses

In Table 2, the univariable cox regression model for Dutch data and Taiwanese data is performed separately by each country, whereas the combined data is analysed at each site using the privacy-preserving federated algorithm. Our findings showed that increasing age, male gender, higher stage, poorer differentiation grade, surgery with adjuvant radiation and/or chemotherapy, and location (e.g. floor of mouth, gum, buccal, and other parts of mouth) were all significant prognostic factors for shorter survival in both the Netherlands and Taiwan. However, period of diagnosis and hospital volume are influential prognostic factors for longer survival in Taiwan, but not in the Netherlands. In combined data, without adjusting for other factors, OCC patients in the Netherlands had worse overall survival than those in Taiwan (HR, 1.39; 95% CI 1.34–1.44). Additionally, the hazard ratio pattern of all prognostic factors, except gender, is similar between individual data and combined data. With regard to gender, Dutch data and Taiwan data show that women's overall survival rate is significantly better than men's; however, in the combined data, because of the higher survival rate of male patients in Taiwan, the survival curve of women crosses the curve of men. Therefore, the HR of gender in the combined data shows no significance (HR, 0.97; 95% CI 0.93–1.01).

**Table 2:** Univariable cox regression analyses.

	Netherlands		Taiwan		Combined	
	HR	95% CI	HR	95% CI	HR	95% CI
**Country**						
Taiwan	–	–	–	–	1.00	–
The Netherlands	–	–	–	–	1.39	1.34–1.44
**Age**						
< 60 years	1.00	–	1.00	–	1.00	–
60–69 years	1.48	1.36–1.62	1.19	1.13–1.24	1.27	1.22–1.32
≥ 70 years	2.55	2.36–2.77	1.93	1.83–2.03	2.16	2.07–2.24
**Gender**						
Female	1.00	–	1.00	–	1.00	–
Male	1.18	1.10–1.26	1.11	1.04–1.19	0.97	0.93–1.01
**Period of diagnosis**						
2004–2007	1.00	–	1.00	–	1.00	–
2008–2011	0.93	0.86–1.01	0.84	0.81–0.88	0.85	0.82–0.89
2012–2016	0.93	0.85–1.01	0.72	0.68–0.75	0.75	0.72–0.78
**Stage**						
Early stage	1.00	–	1.00	–	1.00	–
Advanced stage	2.12	1.99–2.26	3.11	2.99–3.23	2.78	2.69–2.88
Unknown	1.34	0.85–2.11	1.65	1.41–1.92	1.45	1.26–1.67
**Location**						
Other/unspecified parts of tongue	1.00	–	1.00	–	1.00	–
Mucosa of lip	0.81	0.60–1.09	0.99	0.87–1.13	0.95	0.84–1.07
Floor of mouth and gum	1.34	1.25–1.44	1.36	1.29–1.43	1.42	1.36–1.48
Hard palate	1.18	0.89–1.56	1.79	1.60–2.00	1.65	1.49–1.83
Buccal and other parts of mouth	1.29	1.17–1.43	1.07	1.03–1.12	1.04	1.01–1.08
**Grade**						
Well differentiated	1.00	–	1.00	–	1.00	–
Moderately differentiated	1.64	1.48–1.83	1.51	1.45–1.58	1.57	1.51–1.63
Poorly or undifferentiated	2.28	2.01–2.58	2.65	2.49–2.83	2.63	2.49–2.78
Unknown	1.49	1.32–1.69	1.02	0.94–1.10	1.23	1.16–1.31
**Treatment**						
Primary surgery	1.00	–	1.00	–	1.00	–
Surgery with radiotherapy and/or chemotherapy	1.62	1.52–1.73	2.60	2.50–2.70	2.29	2.22–2.36
**Hospital volume (oral cavity surgeries/year)**						
≥ 100	1.00	–	1.00	–	1.00	–
50–99	0.94	0.82–1.07	1.05	0.99–1.09	1.13	1.08–1.17
< 50	0.91	0.81–1.04	1.11	1.05–1.16	1.24	1.20–1.29

The figures in the Netherlands and Taiwan columns are computed locally, while the combined column was computed using the privacy-preserving federated algorithm.

### Multivariable analyses

As shown in Table 3, younger age at diagnosis, female gender, recent years at diagnosis, early stage, well differentiated grade, receiving primary surgery alone, and higher hospital volume were all significant independent prognostic factors for longer survival in the combined data. After adjusting for other prognostic factors, including age, gender, period of diagnosis, stage, location, grade, treatment, and hospital volume, patients with OCC in Taiwan had slightly better outcomes than those in the Netherlands (HR, 1.06; 95% CI 1.01–1.12). Moreover, only patients with hard palate cancers (HR, 1.30; 95% CI 1.17–1.45) had poorer survival after adjusting other covariables. Patients with surgery and adjuvant radiation and/or chemotherapy (HR, 1.40; 95% CI 1.34–1.46) had poorer survival than those with primary surgery alone. Compared with patients treated in the hospitals with ≥ 100 oral cavity surgeries/year, patients treated in the hospitals with < 50 surgeries/year (HR 1.13; 95% CI 1.08–1.08) were independently associated with a poorer survival.

**Table 3:** Multivariable cox regression analyses.

	Netherlands		Taiwan		Combined	
	HR	95% CI	HR	95% CI	HR	95% CI
**Country**						
Taiwan	–	–	–	–	1.00	–
The Netherlands	–	–	–	–	1.06	1.01–1.12
**Age**						
< 60 years	1.00	–	1.00	–	1.00	–
60–69 years	1.49	1.36–1.62	1.29	1.23–1.35	1.31	1.26–1.36
≥ 70 years	2.80	2.58–3.04	2.30	2.18–2.43	2.40	2.30–2.50
**Gender**						
Female	1.00	–	1.00	–	1.00	–
Male	1.27	1.19–1.36	1.23	1.15–1.32	1.23	1.18–1.29
**Period of diagnosis**						
2004–2007	1.00	–	1.00	–	1.00	–
2008–2011	0.93	0.86–1.01	0.82	0.78–0.86	0.85	0.82–0.88
2012–2016	0.87	0.80–0.96	0.67	0.64–0.70	0.73	0.70–0.76
**Stage**						
Early stage	1.00	–	1.00	–	1.00	–
Advanced stage	1.93	1.78–2.10	2.29	2.18–2.41	2.19	2.10–2.29
Unknown	1.46	0.93–2.31	1.57	1.35–1.83	1.46	1.26–1.69
**Location**						
Other/unspecified parts of tongue	1.00	–	1.00	–	1.00	–
Mucosa of lip	0.76	0.56–1.02	1.13	0.99–1.28	1.06	0.94–1.19
Floor of mouth and gum	1.11	1.03–1.20	0.98	0.92–1.03	1.01	0.97–1.06
Hard palate	0.92	0.70–1.22	1.40	1.25–1.57	1.30	1.17–1.45
Buccal and other parts of mouth	1.02	0.92–1.13	1.01	0.97–1.05	1.00	0.96–1.04
**Grade**						
Well differentiated	1.00	–	1.00	–	1.00	–
Moderately differentiated	1.51	1.35–1.68	1.36	1.31–1.42	1.38	1.33–1.44
Poorly or undifferentiated	1.90	1.67–2.15	2.06	1.93–2.20	1.92	1.82–2.03
Unknown	1.50	1.33–1.70	1.10	1.02–1.19	1.24	1.17–1.32
**Treatment**						
Primary surgery	1.00	–	1.00	–	1.00	–
Surgery and radiotherapy and/or chemotherapy	1.04	0.96–1.13	1.52	1.45–1.60	1.40	1.34–1.46
**Hospital volume (oral cavity surgeries/year)**						
≥ 100	1.00	–	1.00	–	1.00	–
50–99	0.90	0.79–1.03	1.00	0.95–1.04	1.01	0.97–1.05
< 50	0.94	0.82–1.07	1.19	1.13–1.25	1.13	1.08–1.18

The figures in the Netherlands and Taiwan columns are computed locally, while the Combined column was computed using the privacy-preserving federated algorithm.

**Figure 1:**
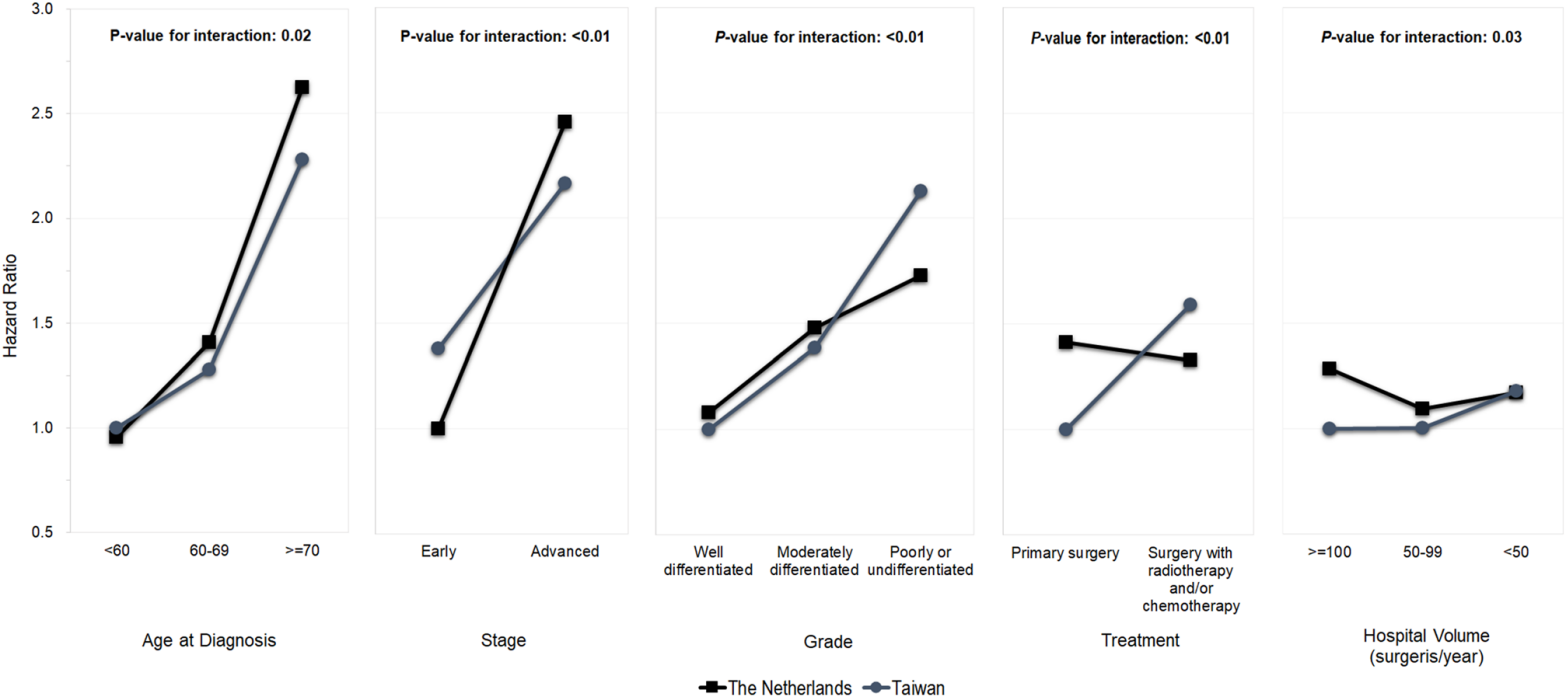
Interaction effects between country and five other prognostic factors.

Prognostic factors with significant factor-by-country interaction are shown in Fig. 1; the following factors have differential effects on survival of OCC patients in the Netherlands and Taiwan: age at diagnosis, stage, tumour grade, treatment, and hospital volume. First, the mortality risk of OCC patients in the Netherlands and Taiwan both increased with increasing age; however, Dutch patients had a stronger association between risk of dying and increasing age than Taiwanese patients. The risk of death for patients younger than 60 years was slightly lower in the Netherlands than in Taiwan, but patients older than 70 years in the Netherlands were at higher risk of death than those in Taiwan. Second, higher stage increased the risk of death of OCC patients in both the Netherlands and Taiwan. However, the risk increments are different in the two countries such that early staged OCC patients had higher risk of death in the Netherlands than those in Taiwan, whereas patients with advanced stage in the Netherlands were at lower risk of death than in Taiwan. Third, the risk of death for patients with well and moderately differentiated grade was slightly lower in Taiwan than in the Netherlands. However, patients with poorly differentiated grade in Taiwan were at higher risk of death than those in the Netherlands. Fourth, OCC patients receiving surgery alone in the Netherlands had much higher risk of death than those in Taiwan; but the risk of death for patients in Taiwan receiving adjuvant radiotherapy and/or chemotherapy was higher than those in the Netherlands. And finally, the mortality risk of OCC in Taiwanese patients who were treated in hospitals with higher hospital volume (≥ 50 surgeries per year) was lower than in Dutch patients. However, patients treated in hospitals with lower hospital volume (< 50 surgeries per year) had similar outcomes in both the Netherlands and Taiwan.

## Discussion

The aetiology of OCC in the Netherlands and Taiwan are different. Although smoking and alcohol consumption are the major risk factors in both countries, betel nut chewing is an important risk factor for Taiwan, which may explain why the incidence rates differ greatly between the two countries. Patient characteristics and experts’ experience with treating this disease may result in survival differences between the Netherlands and Taiwan. Our findings also confirmed the prognostic factors of oral cancer reported in previous studies^26–28^. In the present study, we found that the outcomes of patients treated in the Netherlands and Taiwan were slightly different after controlling for other prognostic factors. As for the potential prognostic factors, we found that age at diagnosis, gender, period of diagnosis, stage, tumour grade, treatment modalities, and hospital volume significantly influence the survival of OCC patients. Five prognostic factors (age, stage, grade, treatment modality and hospital volume) of OCC have differential effects on survival between the Netherlands and Taiwan.

As mentioned previously, we found that the mortality risk of OCC patients, both in the Netherlands and Taiwan, increased with increasing age, higher stage and poorer differentiation grade after adjusting for other prognostic factors in both countries (Fig. 1). Older patients in the Netherlands are at higher risk of death than in Taiwan. Elderly patients may have multiple comorbidities that affect the choice of treatment and tolerance to treatment; therefore, the burden of comorbidity among older OCC patients may be larger in the Netherlands than in Taiwan. Patients with advanced stage and poorly differentiated grade in Taiwan have a higher risk of death than those in the Netherlands. The presence of extranodal extension may be related to the severity of tumour stage and grade, thereby it may influence the prognosis differences in Taiwan and the Netherlands^29^. Meanwhile, extranodal extension in metastatic lymph nodes is an important predictor of regional recurrence and distant metastasis, and it is related to the poor prognosis of OCC^29^. Data on extranodal extension has been collected in Taiwan since 2011 and in the Netherlands since 2015. Additional information on co-morbid conditions and extranodal extension should be considered in future studies.

Surgery alone is the first choice for OCC treatment both in the Netherlands and Taiwan. Surgeons’ experiences, such as complete resection with a tumour-free margin and comprehensive neck dissection, may be critical points in OCC treatment and prognosis. Previous studies in Taiwan showed that patients treated in hospitals with high surgery volume had better OCC survival^30,31^. The risk of death for OCC patients receiving surgery alone in the Netherlands was much higher than in Taiwan, which may be due to differences in surgical experience and/or patient selection. Additionally, previous studies showed that Dutch patients treated in hospitals with different volumes did not differ significantly; this may be due to its highly centralized treatment of head and neck tumours in the Netherlands^32^. However, the mortality risk of OCC in Taiwanese patients who were treated in hospitals with higher hospital volume (≥ 50 surgeries per year) was lower than in Dutch patients; thereby there may still be opportunities for improvement of OCC care in the Netherlands.

Nowadays, the clinical guidelines state that postoperative chemoradiation is recommended for patients with extranodal extension, but is also considered as an alternative to adjuvant radiotherapy for patients with positive surgical margins, pT3 to T4 primary tumours, pN2 to pN3 lymph node disease, perineural invasion, and lymphovascular invasion to improve control rates^33^. Although the risk of death for patients in Taiwan receiving adjuvant radiotherapy and/or chemotherapy was higher than those in the Netherlands, this difference might be explained by unmeasured pathological characteristics, such as resection margins status, extranodal extension, perineural invasion, lymphovascular invasion, performance status and comorbidity. The lack of this information is a limitation of our research. Otherwise, the removal of known risk factors including smoking and alcohol drinking even after diagnosis may reduce the risk of recurrences and second tumours in existing oral cancer patients and also improve the prognosis^34^. However, it is less clear to date how the delays in diagnosis or treatment affect the cancer stage at diagnosis and survival in oral cancer patients^35^. Therefore, these relevant factors, such as lack of individual life-style habits and delays in diagnosis or treatment, should be considered in future research.

Today, only a limited amount of analyses has been developed for the federated infrastructure. For routine use, however, the infrastructure needs to be extended with all commonly used algorithms for data analyses. The main limitation of this work is that the algorithm to check the proportional hazard assumption was not yet implemented. The multivariable regression (18 covariates, Table 3) required a computation time of around 6 min, and the coefficients converged after 5 iterations. Alternative implementations may better deal with higher dimensional data^36^. By design, visual inspection of tables with patient data is not supported. Accordingly, performing federated data analysis will require a different way of working. Advanced quality checking software and adding algorithms for descriptive statistics may mitigate this limitation, as they allow to better understand quality and limitations of datasets^37^.

In the past, combined and interaction analyses of individual patient data from different countries required sharing data between different parties and processing the pooled data in a designated central lab. As it respects patient privacy and complies to data protection regulations, the federated analysis of distributed data platform makes it possible to perform analyses of individual patient data without exchanging patient-level information. To enable this study, we successfully developed an open source IT infrastructure that allows the deployment of algorithms for federated analysis of distributed data and used it for survival analysis for OCCs on patient data from the Netherlands and Taiwan Cancer Registries. This work is the first application of this technology to enable analyses of data from multiple cancer registries. In future work, this infrastructure can be expanded with exploratory analyses and other regression and classification algorithms. Moreover, it can be applied to train artificial intelligence models on multimodal data, including imaging^38,39^. For studies where individual datasets are insufficient (e.g. in international comparisons and studies on rare cancers), the use of a federated infrastructure may become the de-facto standard.

## Data Availability

Data was obtained following the standard data usage request processes at both the Netherlands Cancer Registry and Taiwan Cancer Registry. After approval by the Supervisory Committees, the data were made available from both cancer registries.

## Competing of interest

Dr. van Soest reports personal fees from Medical Data Works B.V., grants from Varian Medical Systems, outside the submitted work; . Dr. Dekker reports grants and personal fees from Varian Medical Systems, personal fees from Medical Data Works, grants from Philips, grants from Mirada Medical, personal fees from Roche, personal fees from Janssen, personal fees from Accuray, outside the submitted work; . Dr. Geleijnse, Dr Chiang, Ms. Schuurman, Mr. Sieswerda, Dr. Lee, Dr Lee and Dr Verbeek have nothing to disclose.
